# Supplementary material for: Nanochemical Cell-Surface Evaluation in Photothermal Spectroscopic Imaging of Antimicrobial Interactions in the Model System Bacillus subtilis and Vancomycin
Source: Anal Chem. 2025 Oct 24;97(43):23914–26. doi: 10.1021/acs.analchem.5c03502 (PMC12590463; doi:10.1021/acs.analchem.5c03502)
Supplement: Supplementary file 1 [file ac5c03502_si_001.pdf]

Supporting Information: Nano-chemical cell-surface evaluation in photothermal spectroscopic imaging of antimicrobial interaction in model system Bacillus subtilis & vancomycin

Maryam Ali,[†,‡] Robin Schneider,[‡,¶] Anika Strecker,[‡,§] Nila Krishnakumar,[‖,‡] Sebastian Unger,[⊥,‡] Mohammad Soltaninezhad,[†,‡] Johanna Kirchhoff,[†,‡,#] Astrid Tannert,[@,‡,#] Katerina A. Dragounova,[†,‡,#] Rainer Heintzmann,[†,‡,‖] Anne-Dorothea Müller,[△] Christoph Krafft,[†,‡] Ute Neugebauer,[†,‡,‖,@,#] and Daniela Täuber[*,†,‡]

†*Institute of Physical Chemistry, Friedrich-Schiller-Universität Jena, 07743 Jena, Germany*

‡*Leibniz Institute of Photonic Technology, Albert-Einstein-Straße 9, 07745 Jena, Germany*

¶*Aalen University of Applied Sciences, Beethovenstraße 1, 73430 Aalen, Germany*

§*Ernst-Abbe University of Applied Sciences, 07745 Jena, Germany*

‖*Abbe Center of Photonics, Friedrich-Schiller-Universität Jena, 07745 Jena, Germany*

⊥*Institute of Physical Chemistry, Friedrich-Schiller-Universität Jena, 07743 Jena Germany*

#*Jena University Hospital, 07747 Jena, Germany*

@*Jena Biophotonics and Imaging Laboratory, 07745 Jena, Germany*

△*Anfatec Instruments AG, Melanchtonstraße 28, 08606 Oelsnitz, Germany*

E-mail: dantaube@gmx.de

Abstract

The Supporting Information provides a short summary of the theoretical framework in photo-induced force microscopy based on the considerations by [Jahng *et al., Phys. Rev. B*, 2022, **106**, 155424] and [Anindo *et al., J. Phys. Chem C*, 2025, **129**, 4517] followed by a discussion of the effect of varying the mechanical detection frequency and examples of scan artifacts.

Contents

PiF-IR signal detection in side band mode **S-3**

Effect of varying mechanical resonance frequency **S-4**

Examples of scan artifacts in PiF-IR **S-6**

PiF-IR signal detection in side band mode

In the following, we will provide a short summary of the photoinduced force (F_{pi}) in photoinduced force microscopy (PiFM, including PiF-IR) operated in heterodyne side-band mode. PiFM evaluates the gradient of the distance-dependent interaction force F_{ts} between the AFM tip and the sample. A general expression for F_{ts} is given by[S1–S3]

$$F_{\text{ts}}(z) = F_{\text{c}}(z) + F_{\text{nc}}(z) \tag{1}$$

where $F_{\text{c}}(z)$ describes the contribution from conservative and $F_{\text{nc}}(z)$ non-conservative forces and z is the direction normal to the sample plane. The conservative forces are given by

$$F_{\text{c}}(z) \approx \begin{cases} \frac{-H_{\text{eff}} r}{12 z^2} & \text{for } (z > r_0) \\ \frac{-H_{\text{eff}} r}{12 r_0^2} + \frac{4}{3} E^* \sqrt{(r_0 - z)^3 r} & \text{for } (z < r_0) \end{cases}$$

H_{eff} is the effective Hamaker constant which is a measure for the strength of the interaction energy between two bodies via a medium, r is the tip apex radius and E^* is the effective Young's or elastic modulus which is a measure of the stiffness and rigidity of the material. When the distance between the tip and the ample is less than the interatomic distance r_0, the conservative force becomes the sum of two forces. The first term is the non-contact van der Waals force and the second term is the contact Derjaguin, Muller, and Toporov (DMT) force.[S2]

In the heterodyne detection mode, F_{pi} is detected in non-contact AFM mode, i.e. employing a high setpoint of the cantilever oscillation and a small oscillation amplitude $A_2 \approx 1 - 2$ nm for the oscillation driven at the second mechanical resonance frequency f_2 of the cantilever. F_{pi} is obtained at the first mechanical resonance frequency f_1, and the illuminating laser is modulated at $f_m = f_2 - f_1$.[S2,S3] In the small oscillation limit, F_{pi} can be given as[S3,S4]

$$F_{\text{pi}} \approx \frac{\delta F_{\text{ts}}}{\delta z} \Delta z, \tag{2}$$

where F_{ts} is the distance-dependent interaction force between the AFM tip and the sample and Δz is the photoinduced thermal expansion normal to the sample plane.

Effect of varying mechanical resonance frequency

It is well known that mechanical properties such as stiffness and viscosity influence the phase as well as the resonance frequencies in dynamic AFM measurements.[S5] A change in these properties can be recognized by a corresponding phase shift between the driving oscillation and the resulting oscillation of the cantilever. Such shifts are presented in the AFM phase-contrast images of the scanned sample areas.

In our first two series of PiF-IR measurements, the mechanical resonance frequency of the system was evaluated once prior to scanning a sample area for PiF signal acquisition. As a result, the strength of the recorded PiF signal is modulated by the distance to the actual

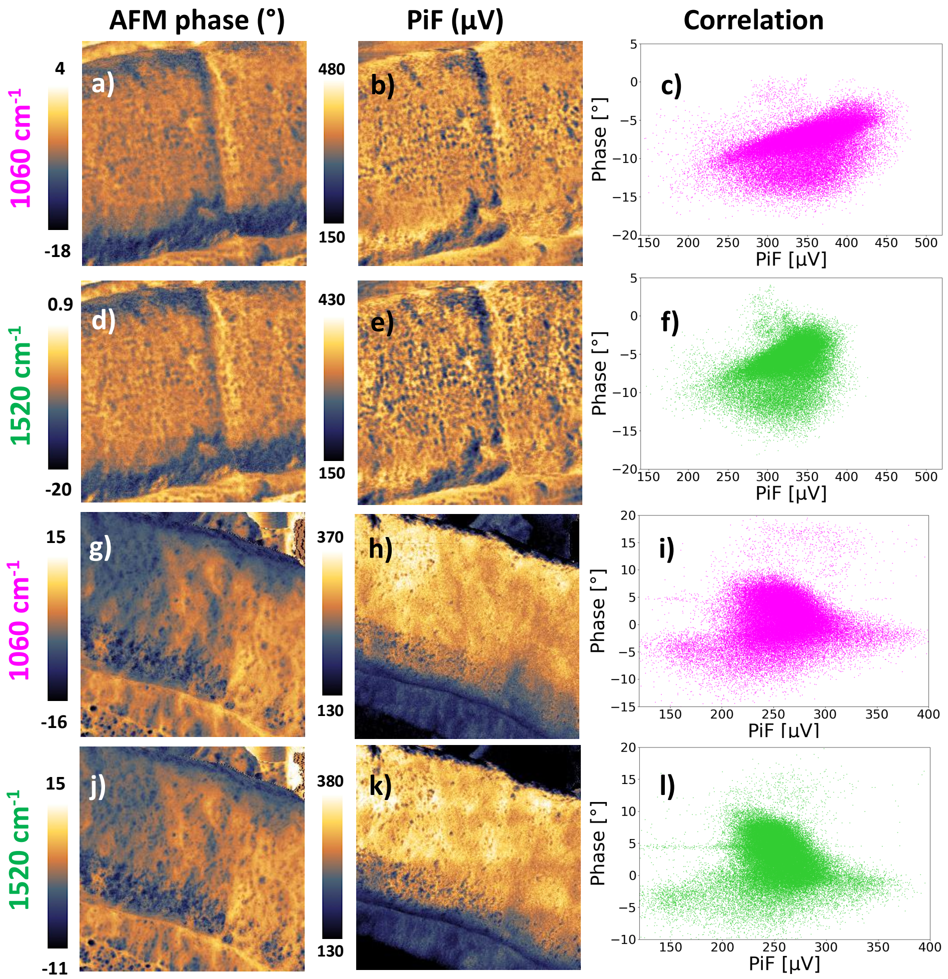

Figure S1: **Correlations between AFM phase and PiF contrasts of treated *B. subtilis* harvested after 15 min.** a-c) and d-f) scan of cell of series 2 acquired @1060 cm^{-1} and @1520 cm^{-1}, respectively. g-i) and j-l) scan of cell of series 3 acquired @1060 cm^{-1} and @1520 cm^{-1}, respectively. AFM phase images: a,d,g,j, PiF contrasts: b,e,h,k and their pixel-wise correlations: c,f,i,l.

resonance frequency at the particular position in the scan. As the AFM phase shifts together with the resonance frequency, this can be seen by comparing the acquired PiF contrasts with the AFM phase contrasts in the same position. An example is given in Figs. S1a-f showing AFM phase and PiF contrasts of two subsequently acquired high-resolution scans of a treated *B. subtilis* cell harvested a 15 min from our series 2 together with pixel-wise correlations of PiF and AFM phase. For both illumination frequencies, a predominately linear correlation is seen between PiF intensities and the simultaneously acquired phase phase of the cantilever

oscillation. In contrast, a linear correlation is not observed in the corresponding data sets of our series 3, which were acquired with an adjustment of f_1 in each pixel prior to acquisition (Figs. S1g-l).

Examples of scan artifacts in PiF-IR

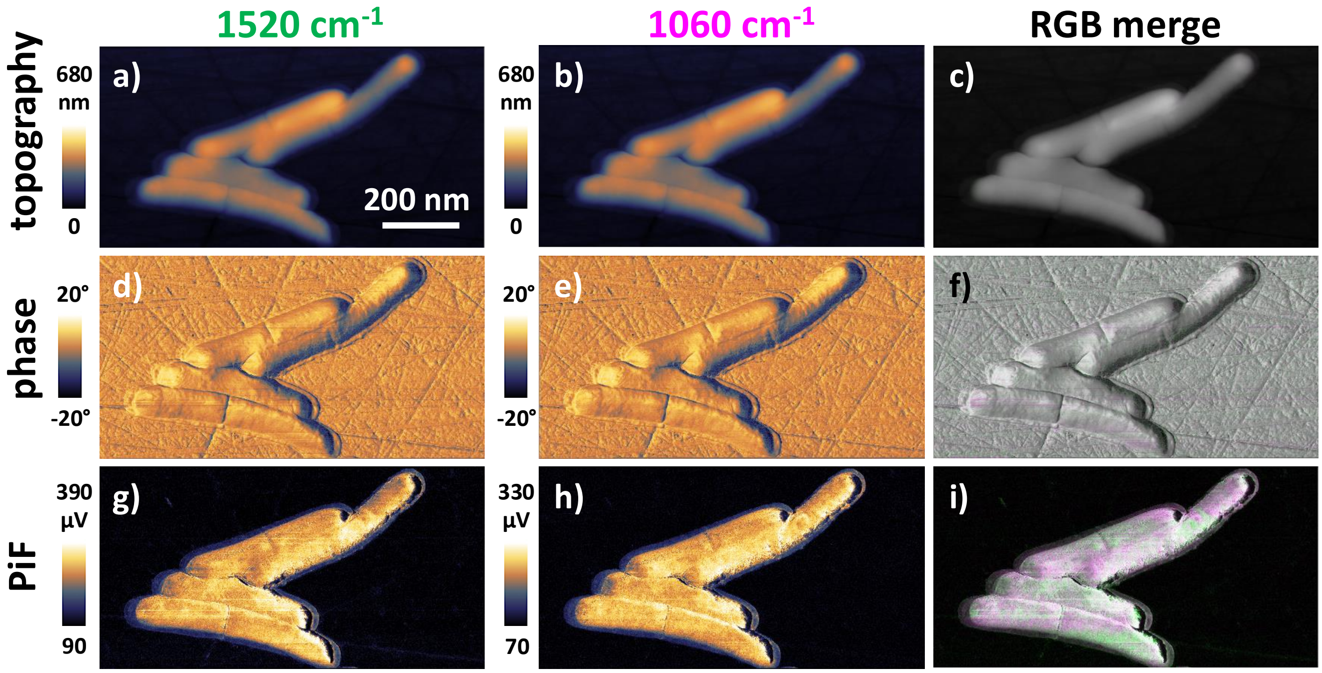

Figure S2: **Subsequently acquired PiF contrasts of untreated *B. subtilis* harvested after 30 min, series 3 together with RGB merges**. a-c) AFM topography, d-f) AFM phase and g-j) PiF acquired @1520 cm^{-1} (a,d,g) and @1060 cm^{-1} (b,e,h).

In AFM several types of artifacts are known to appear.[S6–S8] An example is sample material attached to the AFM probe during scanning. This typically results in either a recurring pattern appearing in the image in the case of a particle adhered to the tip, or in lines in the fast scanning direction in the case of loose materials temporarily attached to the tip. The latter was observed in subsequent scans of the untreated *B. substilis* cells harvested after 30 min from our series 3, see Fig. S2. Horizontal lines appear in the AFM phase and in the PiF contrasts of both scans but not in the lower-resolution topography images (acquired at the second mechanical resonance frequency). As discussed in the methods section, over-

shooting of the tip oscillation at the steep edge of bacteria cells may cause tip-sample contact even in dynamic non-contact AFM. Obviously, this occurred during the first scan at 1520 cm^{-1} resulting in sharp horizontal lines in the AFM phase and in the PiF contrast of the scan; see Figs. S2d and g, respectively. Several of these lines also appear in the subsequent scan at 1620 cm^{-1} (Figs. S2e and h), which shows that the material was not only temporarily attached to the tip, but also smeared over the surface of the sample during scanning. Due to the high surface sensitivity of PiF-IR even small amounts of material smeared over the surface show pronounced effects on the signal intensity, as can be seen in the RGB merge (Fig. S2i) of the two subsequently acquired PiF contrasts. However, the lines of the smeared material are narrow and therefore could be avoided for the acquisition of PiF-IR spectra on that sample.

In PiF-IR scanning artifacts may also be introduced by instabilities in the alignment of the laser beam illuminating the tip-sample region of by power instabilities of the light source. In some cases, we had observed feedback from the cooling system on the PiF intensity in our instrument. An example is presented in Fig. S3 showing subsequent scans of a treated *B. subtilis* harvested after 15 min from our series 2. The periodic instability in signal intensity resulted in a pattern of equidistant horizontal stripes in single PiF contrasts (Figs. S3). It is quite unlikely that the next scan will start in the same phase of the periodic signal. Therefore, the pattern differs in subsequent scans. However, such a broad horizontal stripe pattern can be easily corrected for by a line-wise correction using the mean intensity of each line, a feature frequently used to improve scanning probe microscopy images. Figs. S3j, k and i show the PiF contrasts after the application of this correction for the subsequent scans and the RGB merge, respectively. The corrected images improve the chemical contrast, which was already visible in the raw images. In this particular sample position, the PiF contrast appears to be predominantly perpendicular to the fast scanning direction, and therefore is not affected by this kind of image processing. In case of a chemical contrast parallel to the fast scanning direction, a similar image processing could cause a conflict with the signal of

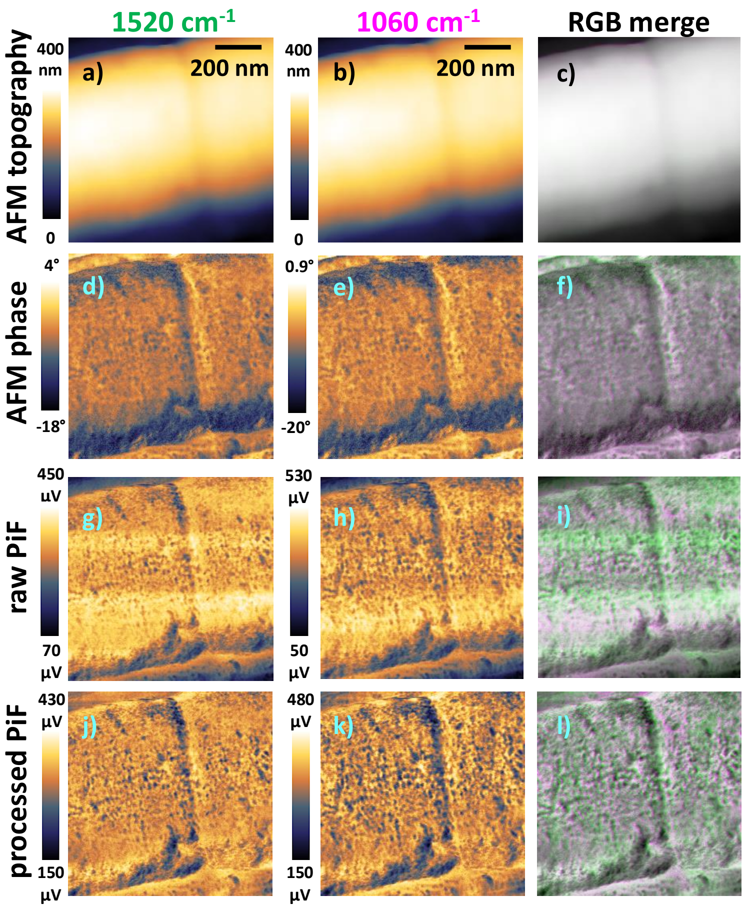

Figure S3: **PiF signal instability in subsequently acquired PiF contrasts of treated *B. subtilis* harvested after 15 min, series 2, together with RGB merges**. a-c) AFM topography, d-f) AFM phase, g-j) acquired PiF and j-l) line wise corrected PiF acquired @1520 cm^{-1} (a,d,g,j) and @1060 cm^{-1} (b,e,h,k).

interest.

References

(S1) Sifat, A. A.; Jahng, J.; Potma, E. O. Photo-induced force microscopy (PiFM) – principles and implementations. *Chemical Society Reviews* **2022**, *51*, 4208–4222, Publisher: The Royal Society of Chemistry.

(S2) Jahng, J.; Kim, B.; Lee, E. S. Quantitative analysis of photoinduced thermal force: Sur-

face and volume responses. *Physical Review B* **2022**, *106*, 155424, Publisher: American Physical Society.

(S3) Anindo, S. T.; Täuber, D.; David, C. Photothermal Expansion of Nanostructures in Photoinduced Force Microscopy. *The Journal of Physical Chemistry C* **2025**, *129*, 4517–4529, Publisher: American Chemical Society.

(S4) Jahng, J.; Potma, E. O.; Lee, E. S. Nanoscale spectroscopic origins of photoinduced tip-sample force in the midinfrared. *Proceedings of the National Academy of Sciences of the United States of America* **2019**, *116*, 26359–26366, Publisher: National Academy of Sciences.

(S5) Bian, K.; Gerber, C.; Heinrich, A. J.; Müller, D. J.; Scheuring, S.; Jiang, Y. Scanning probe microscopy. *Nature Reviews Methods Primers* **2021**, *1*, 36, Publisher: Nature Publishing Group.

(S6) Ricci, D.; Braga, P. C. In *Atomic Force Microscopy: Biomedical Methods and Applications*; Braga, P. C., Ricci, D., Eds.; Humana Press: Totowa, NJ, 2004; pp 25–37.

(S7) Ukraintsev, E.; Kromka, A.; Kozak, H.; Remeš, Z.; Rezek, B. In *Atomic Force Microscopy Investigations into Biology - From Cell to Protein*; Frewin, C., Ed.; InTech Europe: Rijeka, Croatia, 2012.

(S8) Canale, C.; Torre, B.; Ricci, D.; Braga, P. C. *Methods in Molecular Biology*; Humana Press Inc., 2011; Vol. 736; pp 31–43, ISSN: 10643745.
